# Modelling, additive layer manufacturing and testing of interlocking structures for joined components

**DOI:** 10.1038/s41598-022-06521-z

**Published:** 2022-02-15

**Authors:** Gonzalo Peralta Marino, Stefano De la Pierre, Milena Salvo, Andrés Díaz Lantada, Monica Ferraris

**Affiliations:** 1 Mechanical Engineering Department, Universidad Politécnica de Madrid, Madrid, Spain; 2 DISAT-Department of Applied Science and Technology, Politecnico di Torino, Turin, Italy

**Keywords:** Engineering, Materials science

## Abstract

In this study, authors explore the application of modelling and additive layer manufacturing (ALM) for creating and testing materials with interlocking structures aimed to reduce the stress concentration along the edges of a typical lap joint. The effectiveness of this approach is discussed by means of modelling and experimental validation of joints with interlocking structures obtained by ALM. Considering the achieved results, ALM of interlocking structures constitutes an interesting alternative or complement to traditional joining processes, as it may help to minimize stress mismatches in the joining region. It may also prevent the use of adhesive or joining post processes, because the joint is created together with the joined components.

## Introduction

In order to join dissimilar materials, especially when they widely differ on micro-structure and properties, the most common technique used is adhesive joining, which is mostly used nowadays due to its favourable cost/efficiency rate, lightness and structural efficiency of the joined components. The most common configuration to test adhesive joints is the “lap joint” one, either single lap or single lap offset, due to ease of fabrication and test efficiency: a lap joint consists of two materials (known as adherends) joined by a third material, the adhesive. Depending on where the joint failure occurs, several failure types can be defined: cohesive failure, which is the most desirable one, when the failure occurs within the adhesive layer; adhesive failure, when adhesive forces between the adherend and the adhesive are weaker than cohesive forces inside the substrate and the adhesives: the adhesive failure is thus located at the interface between adherend and adhesive. It is also possible that the failure occurs partially along both adherends and adhesive: in this case there is either a substrate failure, i.e. it is the adherend to fail first, due to the strength of the adhesive used; or a mixed failure mode, where the failure occurs as a mix between the failure modes mentioned above.

Whatever the failure mode, the maximum load for a lap joint is a complex combination of factors such as the mechanical and chemical properties of materials involved, the joint geometry (length, surface roughness, porosity, surface preparation, etc.), joint thickness, presence of internal strains and the loading mode (tensile, cleavage, tensile-shear, compression-shear, torsion and/or peel).

Depending on the combination of factors described above, different stresses are present along the overlap length in a given joint^1^. The selected lap joint for this work is a Single Lap Offset (SLO) to be loaded in compression: it is well known in the literature^2^ that the shear and normal stress distribution has two high stress peaks at the edges of this joint. The Goland and Reissner’s model has been used for this study as non-linear solution to model high stress peaks at the edges of SLO joints^1,3,4^: it is the classical approximate analysis used and has been found to be the most accurate one when predicting stresses along the lap joint. Other authors have developed other predictive models for the same problem, such as Bigwood and Crocombe^5^, Wang and Zhang^6^ and Luo and Tong^7,8^, within others. Since lap joints failure is mainly due to high stress peaks on the edges of the joint, some works are proposing additive layer manufacturing (ALM) to improve lap joint strength, such as multi-material print-forming^9,10^.

ALM consists of fabrication in a layer-by-layer mode, following geometries from digital models. This layered manufacturing approach allows for the fabrication of multi-material products with complex shapes. Since the fabrication is done by layers, ALM unlocks the fabrication of high complexity components that would be infeasible or highly expensive if done by other more traditional fabrication methods. Recent reviews on ALM (or additive manufacturing technologies AMTs) have put forward current materials, methods, potentials and challenges in the field^11,12^, with a special focus on fused deposition modeling^12^, which stands out for its transformative potential in many industries, thanks to being affordable and widespread. Among well-known challenges, the anisotropy of ALM processes, which affects mechanical stability of 3D printed constructs, stands out.

Reflecting on the above, the simplest and most widespread additive manufacturing method, in many cases just referred to as “3D printing”, is fused deposition modeling (FDM) or fused filament fabrication (FFF), which commonly employs polylactic acid (PLA) or acrylonitrile butadiene styrene (ABS) thermoplastic filaments. The method consists of feeding a filament of material through a heated needle which fuses the thermoplastic material and allows for desired 2D and 3D deposition to create complex objects. Although the cheaper FFF versions employ standard thermoplastics, the more professional systems may employ technical polymers (i.e. PEEK) and thermoplastics filled or reinforced with metallic and ceramic particles or glass and carbon fibres, among others. The layer-by-layer approach leads to anisotropic material properties, as in several additive manufacturing processes^13^.

There are methods that combine *both* the additive manufacturing *and* the multi-material bonding, creating transitions between materials or certain geometries on the adherends surfaces to obtain improved joints^14,15^. Adhesive joining can be complemented or substituted by these methods. Furthermore, ALM development has now made possible some multi-material fabrication processes, by combining two or more dissimilar materials in the same layer in a sequential or almost simultaneous way^16^, hence creating a heterogeneous interface with combined properties.

An example of the implementation of ALM to the joining process is the study reported by Chait^10^, where a new method for designing boundaries between dissimilar materials to better resist common failure modes is reported. This method focused on creating a material gradient such that properties do not present an abrupt transition.

By additive layer manufacturing it is also possible to generate a gradual transition between two materials, obtaining the desired property variation, by making functionally graded materials (FGM) characterized for a compositional gradient from one material to the other^17,18^. FGM are everywhere in nature, where materials do not present an abrupt transition. Instead, materials are perfectly combined to attain an adequate strength and avoid structural failure under working conditions^19^.

In direct connection with the additive manufacture of functionally graded materials, the creation of “3D Matter Made to Order”^20^ and the use of “voxellated matter”^21^, have been also proposed, more as a way of creating constructs with spatially controlled properties from the design stage, than as a method for joining dissimilar materials and components. However, to further expand the industrial applications of these methods and technologies, the modeling of ALM constructs and systematic studies for comparing their potentials with the actual performance of conventional joining procedures, should be undertaken.

The novelty of this work is to *combine finite element modelling and ALM to surface geometry modification necessary to decrease high stress peaks in single lap offset joints*; FEM is used to model different geometries for the surfaces of the two materials to be joined, ALM to obtain the modelled structures, and the experimental validation is done on Single Lap Offset (SLO) joints in compression mode. To the best of authors’ knowledge, this combination has never been reported.

## Materials and methods

### Design, modeling and manufacturing methods

**Figure 1:**
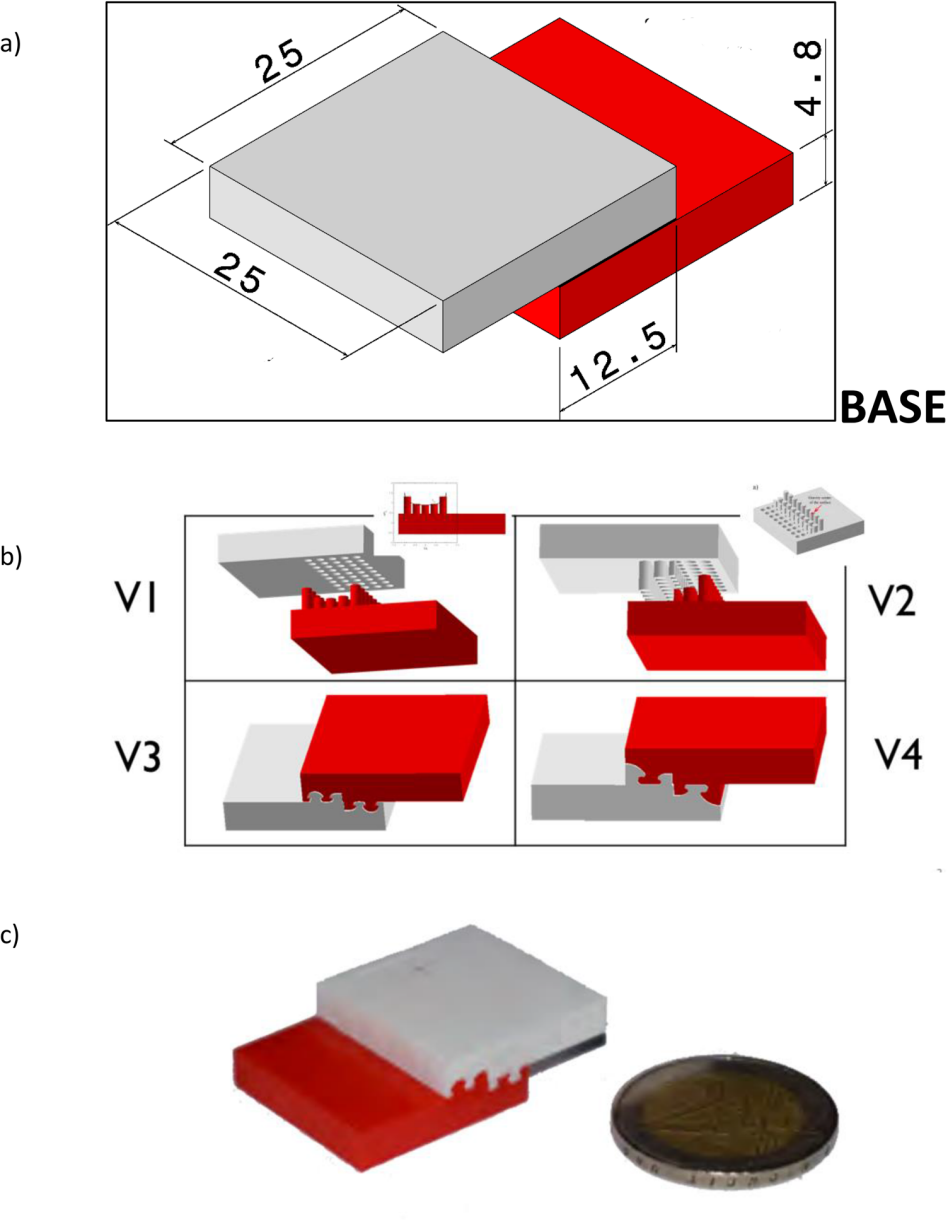
Single lap offset (SLO) joints modelled in this work: (**a**) “Base” is the reference joint, without modification in the joined region (size in mm). (**b**) V1–V4 are joints with the same size as the “base” one, with the joined region modified by ALM to minimize stresses. (**c**) Example of 3D printed prototype, in this case corresponding to V3.

Catia v.5 (Dassault Systèmes) is employed as design and modeling software for performing computer-aided design (CAD) operations and finite-element modeling (FEM) tasks (also referred to as finite-element analyses—FEA). Four designs labelled as V1, V2, V3, V4, and the base one, i.e. a SLO with flat surfaces, are developed through computer-aided design (CAD). The designs are shown in Fig. 1. Designs V1–V4 include interlocking elements forming functional gradients, whose detailed views are also included in Fig. 1.

The V1, V2, V3, and V4 are prototyped by ALM by using the Sakata silver PLA 850, from Sakata ALM filaments (Polimersia Global S.L., Spain) (referred to as *Sakata* in the text) and the Smartfil PLA Cobalt, from Smart Material ALM materials (Spain) (referred to as *Smartfil* in the text). A commercial double extrusion fused deposition modelling machine, BCN 3D Sigma system (BCN 3D Technologies, Casteldefells, Barcelona, Spain), is employed as ALM system. Test probes are printed layer by layer and using sequentially both printing materials in each layer, to obtain the prototypes already assembled in testing position, *without any joining material in between*. Recommended printing parameters according to the materials data sheets are employed, which include: printing temperature of 210 °C for both materials, a heated printing bed at 40 °C, printing speed of 20 mm/s for the initial layers and 40 mm/s for the intermediate and upper layers of the constructs, and layer height of 200 μm. An infill of 100% is applied when generating the slices with the Ultimaker Cura software, so as to work with bulk materials and promote adhesion within and across layers.

Their mechanical behaviour is studied with the support of FEM modeling capabilities of the design software (Catia v.5). In general, for meshing the three-dimensional test probes, linear tetrahedrons are employed as isoparametric solid elements. The automated mesh generation features of the software are employed and mesh size is smoothed until at least three elements are present across each of the interlocking geometries under study. This normally leads to elements of c.a. 0.25 mm, which the authors consider enough for recapitulating the actual designed geometries, especially considering that the extruded filament thickness is around 400 μm and that the layer height is close to 200 μm. Materials properties (Table 1) according to data sheets are added to the library of materials and the loading and boundary conditions for the simulations replicate those from the mechanical testing, as it will be discussed below. Materials in the study are considered isotropic the values of density, Young’s modulus and Poisson ratio are taken into account. Yield strength is considered for analyzing the performance of the joint and predicting failure, for which the Von Mises criterium is applied.

**Table 1:** Properties of materials used in this work^22^.

	Density (g/cm^3^)	Yield strength (MPa)	Young modulus (MPa)	Poisson ratio	Color
Sakata silver PLA850	1.24	50	2315	0.36	White/grey
Smartfil PLA cobalt	1.24	110	3309	0.36	Red/blue

### Mechanical testing

Single lap offset tests in compression load were done by using a universal testing machine (SINTEC D/10): the crosshead speed was set to 1 mm/min. A first group of experiments consisted of testing in compression all the V1–V4 prototypes (without any adhesive in between). For this first set of experiments, without adhesive, prototypes are tested as printed, just after manual cleaning and polishing for eliminating printing debris.

A second group of experiments consisted in the adhesive joining of V4 used to be compared to an adhesive joined, non-modified, base sample; in this case, adherends were firstly manufactured by ALM with Sakata, acetone cleaned then joined using a bi-component epoxy adhesive EPX DP490 (3M Scotch-Weld) supplied as a paste. A thin layer of adhesive was manually placed between the surfaces to be joined, then cured at room temperature for seven days, according to the adhesive datasheet^23^. More information on the mechanical characterization this adhesive is reported in a previous study^24^.

## Results and discussion

### Main results and discussion

Additive layered manufacturing enables the creation of complex geometries and promotes three-dimensional control over materials from the design stage. These resources were used to obtain prototypes (V1–V4), which are characterized by the incorporation of complex interlocking elements designed to modify the joined surfaces, in order *to change the nature of the stresses present along the bond length, aiming at modifying the stress fields and minimizing stress concentrations*.

For the ideation of the interlocking elements, the construction piles theory, which states that “*the reaction forces acting over the piles surface are stronger as the pile sinks deeper into the soil*” is employed as basic design principle^25^. Besides, the precision and resolution of the ALM system are considered in this design for additive manufacturing strategy: different concepts of interlocking geometries are initially considered, but only those technically viable from the manufacturing perspective are further modelled, prototyped and tested.

Thus, by creating a group of pins on the adherends surface (Fig. 1, V1–V4), the shear forces are ideally replaced by the normal ones, which are situated along the surfaces of the pins. Tensile forces are then transmitted from one material to the other through the interface forces between both components along the pins surface. Regarding the pins’ length, they are designed based on the single lap offset (SLO) test shear stress diagram in compression^2^, as shown in Fig. 1 (V1): the higher the normal stress, the higher the pin needed for that section.

Moreover, further piles concepts are modelled and realized. Since there are higher stress concentrations close to the lap edges, authors hypothesize that it may be possible to redistribute the normal stress within the components. The piles are therefore located on the half overlap closer to the gravity centre of each of the components’ surface (Fig. 1, V2). Thereby, the normal forces closer to the overlap edge of one of the components are transmitted to the other through the piles at a point further from its edge, and vice-versa. In addition, piles act also as a support for the shear forces, aiming to reach the failure at higher loads.

Following the same reasoning of the previous design, it is possible to redistribute the stress derived from the peeling stress on the components, with the aim of increasing the joint strength. In consequence, an innovative hook-based design is created, which aims to be subjected to shear loads on the shanks and to tensile loads on the flukes. Two versions are modelled: the first one, V3 in Fig. 1 (V3), which has the main hook geometry on the middle of the overlap; and a second version, V4 in Fig. 1 (V4), which has the main hook geometry on the edge of the overlap.

**Figure 2:**
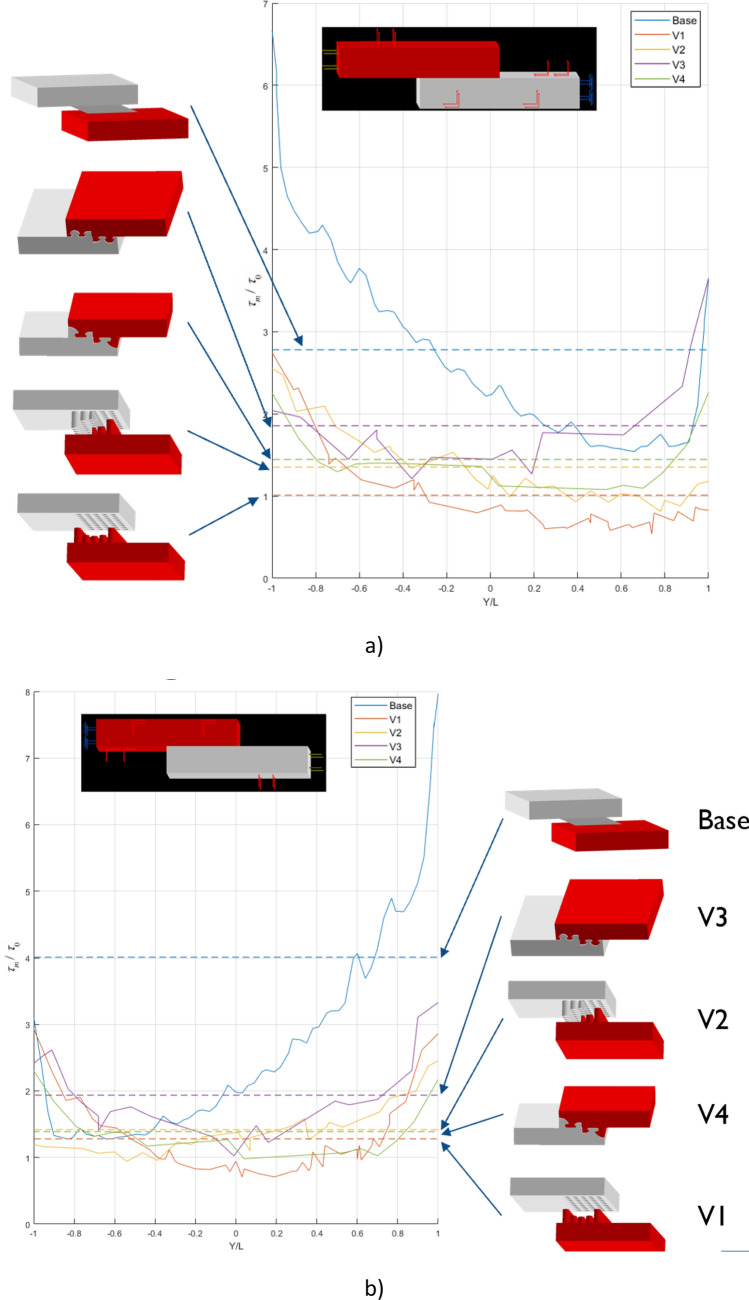
Shear stress distribution along the bond line obtained through FEA of Single Lap Offset (SLO) joints studied in this work, tested in compression: “base” is the reference joint, without modification in the joined region. V1–V4 are joints with the same size of the “base” one, with the joined region modified by ALM where stresses are higher. Load applied on the red (**a**) or on the grey component (**b**). V1, V2 and V4 gave the most uniform stress distribution along the bond line, while the “base” showed the highest one, as expected.

Once the interlocking structures are designed, FEM capabilities are used to compare the different designs and select the most adequate ones for further testing. Figure 2 shows the shear stress distribution along the bond line obtained through FEA of Single Lap Offset (SLO) joints loaded in compression: “base” is the reference joint, without modification in the joined region. V1–V4 are joints with the same size of the “base” one, with the joined region modified by ALM where stresses are higher. Compression load is applied on the red (a) or on the grey component (b).

According to simulation results, V1, V2 and V4 gave the most uniform stress distribution along the bond line, while the “base” showed the highest one, as expected. Figure 2 shows the improvement obtained from all the V1–V4 geometries proposed, since the peak values for the shear stress, which are the points where the failure of the will start, *are significantly reduced*. In addition, the mean values for all the shear stresses were also represented (dashed lines in Fig. 2), from which it is possible to conclude that *all the geometries developed not only reduce the peak values, but also reduce the mean shear stress along the overlap length, and thus increase their strength under compressive forces*.

**Figure 3:**
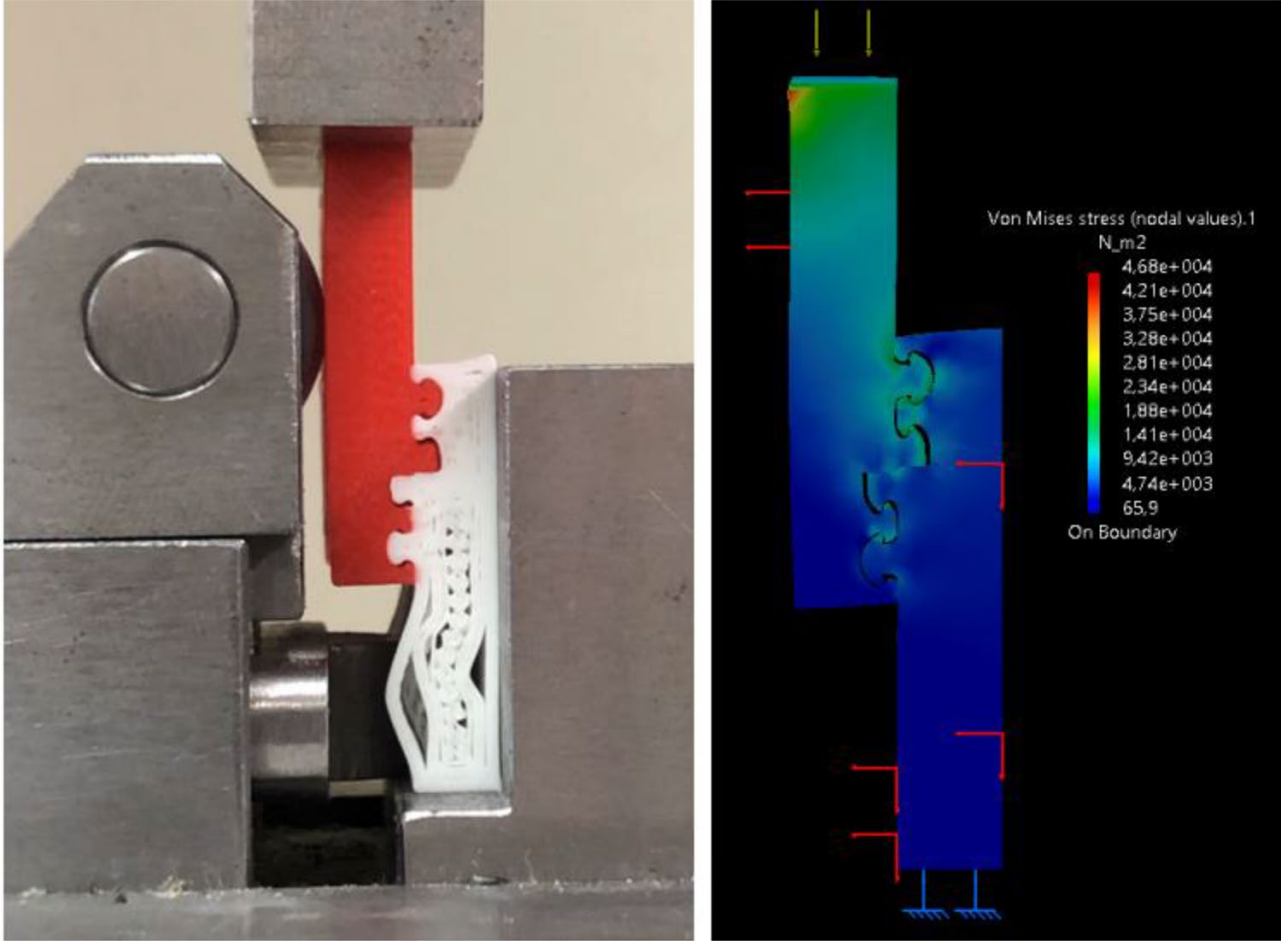
V3 SLO experimental test in compression with failure of the adherend. Mechanical test (left) vs. FEM simulation (right).

The results obtained by modelling have been tested and experimentally validated: a first group of experiments consisted of testing in compression all the V1–V4 prototypes (without any adhesive in between) and the typical result is shown in Fig. 3 for V3. The failure of one of the two adherends under compression load (Fig. 3) without fracture in the joined region always occurred in this test campaign on V1–V4 samples.

From the mechanical tests carried out on all the V1–V4 samples, it can be concluded that, for all these configurations, the strength of one of the adherends (Sakata or Smartfil) is significantly lower than that of the joined area, *even without adhesive in between*. This is a problem intrinsic to most mechanical systems developed using ALM and, more specifically, fused-deposition modelling: the layered process leads to anisotropies and the final mechanical performance depends on the adhesion between deposited layers. In this case, the adhesion between layers of deposited polymeric filament is lower than the strength of the interlocked region, with or without adhesive. This should be considered when designing for additive layered manufacturing.

**Figure 4:**
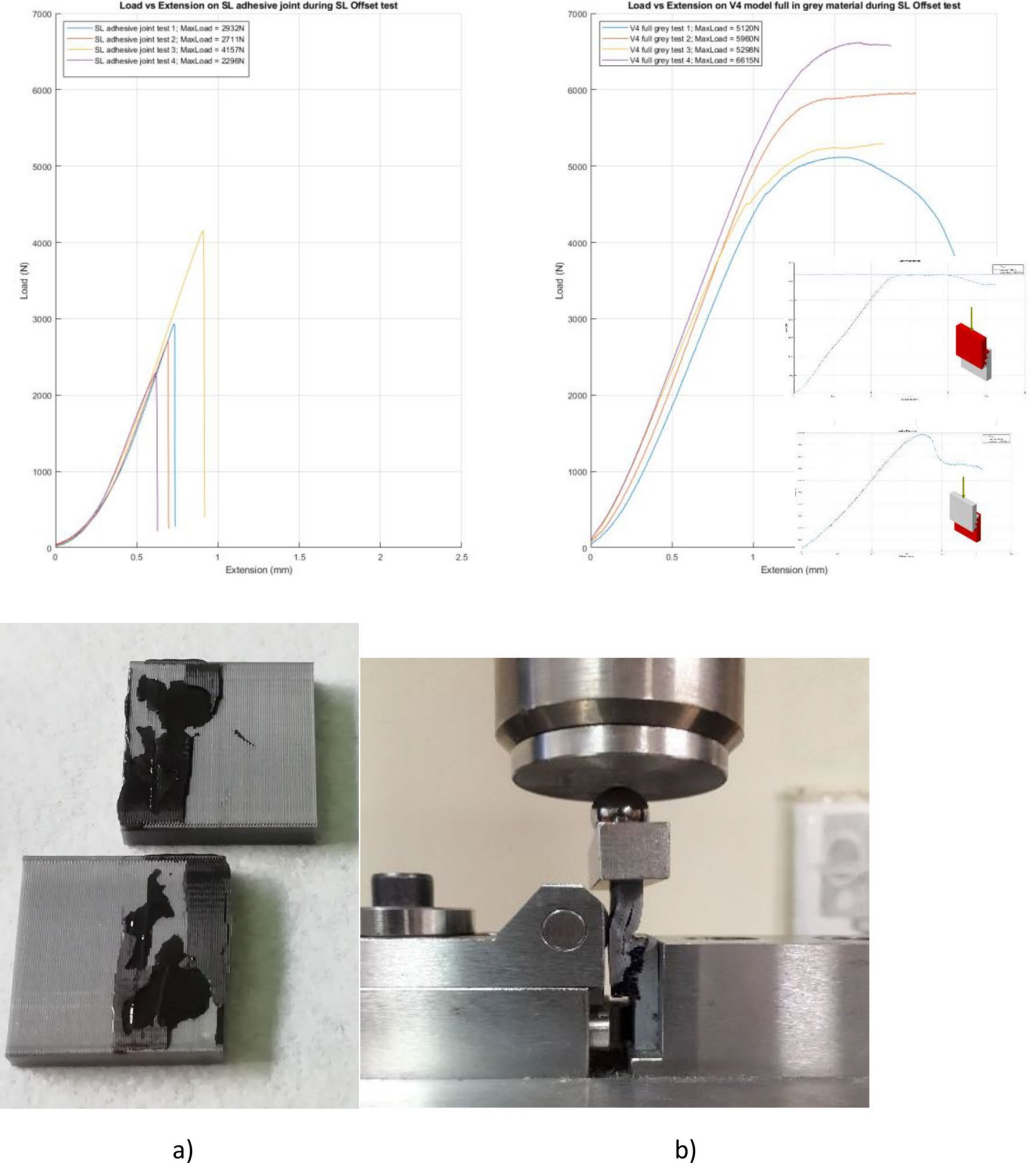
Load/displacement curves of lap shear stress tests results on Sakata joined components: “base” reference joints, without modification in the joined region, joined by *adhesive* and showing brittle failure (**a**); V4 joints, joined by *the same adhesive*, after stop of mechanical test without failure (**b**). (See inset) for comparison purposes, mechanical test result for V4 configuration obtained without adhesive in between.

In order to analyse the potential synergies between interlocking structures and the use of adhesives, a second group of samples was prepared and tested. Sakata material was used to prepare similar joints with base and V4 geometry, chosen for its best performance. In both cases, the two parts to be joined were separately fabricated by ALM and then joined by the epoxy adhesive EPX DP490 (3M Scotch-Weld) (Fig. 4).

The load/displacement curves demonstrated that the “base” joints, without modification in the joined region have a brittle failure, Fig. 4a, ranging from 2200 to 4100 N, corresponding to 7–12 MPa, calculated by dividing the maximum recorded force by the joined area (25.0 mm × 12.5 mm, as in Fig. 1). This apparent shear value is consistent with values reported on the adhesive data sheet from the producer (1.5–5.0 MPa for joining of different plastics)^23^. The torsional shear strength together with a comprehensive mechanical characterization of this adhesive have been reported elsewhere^24^.

According to the datasheet, this bi-component epoxy adhesive is a “*black, thixotropic, gap filling adhesive, designed for use where toughness and high strength are required*”. However, a brittle behavior has been reported in^26^ after lap-shear test on joined glass slabs: all joints failed with a brittle failure starting inside the adhesive and propagating inside the glass. No plastic deformation was measured for this adhesive and its average lap-shear strength was about 19 MPa; however, as the authors correctly pointed out, this value is referred to the “*adhesive shear strength governed by glass failure*”. This is a typical problem arising with lap-shear tests where singularities due to the sample geometry induce stress concentration thus causing premature failure of the adherend.

In our work, the brittle behavior of this adhesive has been clearly demonstrated without any contribution due to the substrates’ failure, as evident in Fig. 4a, which shows the fracture surfaces and the load displacement curves obtained after mechanical test of the base configuration, adhesively joined without modification of the joined region. On the other hand, V4 joints, joined by the same epoxy adhesive, required to stop the mechanical tests *without recording any failure*, Fig. 4b, at values of 5000–7000 N: a non-brittle behaviour of the curves can be noticed, due to the adherend failure in compression.

It is worth noting that the same V4 configuration obtained with Smartfil and Sakata, but without adhesive in between, gave much lower mechanical strength than V4 obtained with Sakata and adhesive; also in these cases it was necessary to stop the mechanical tests *without recording any failure*, (Fig. 4b, see insets), but at values of 1000–3200 N: this was verified both when applying compression load either on Smartfil or on Sakata V4 (Fig. 4b, see insets).

**Figure 5:**
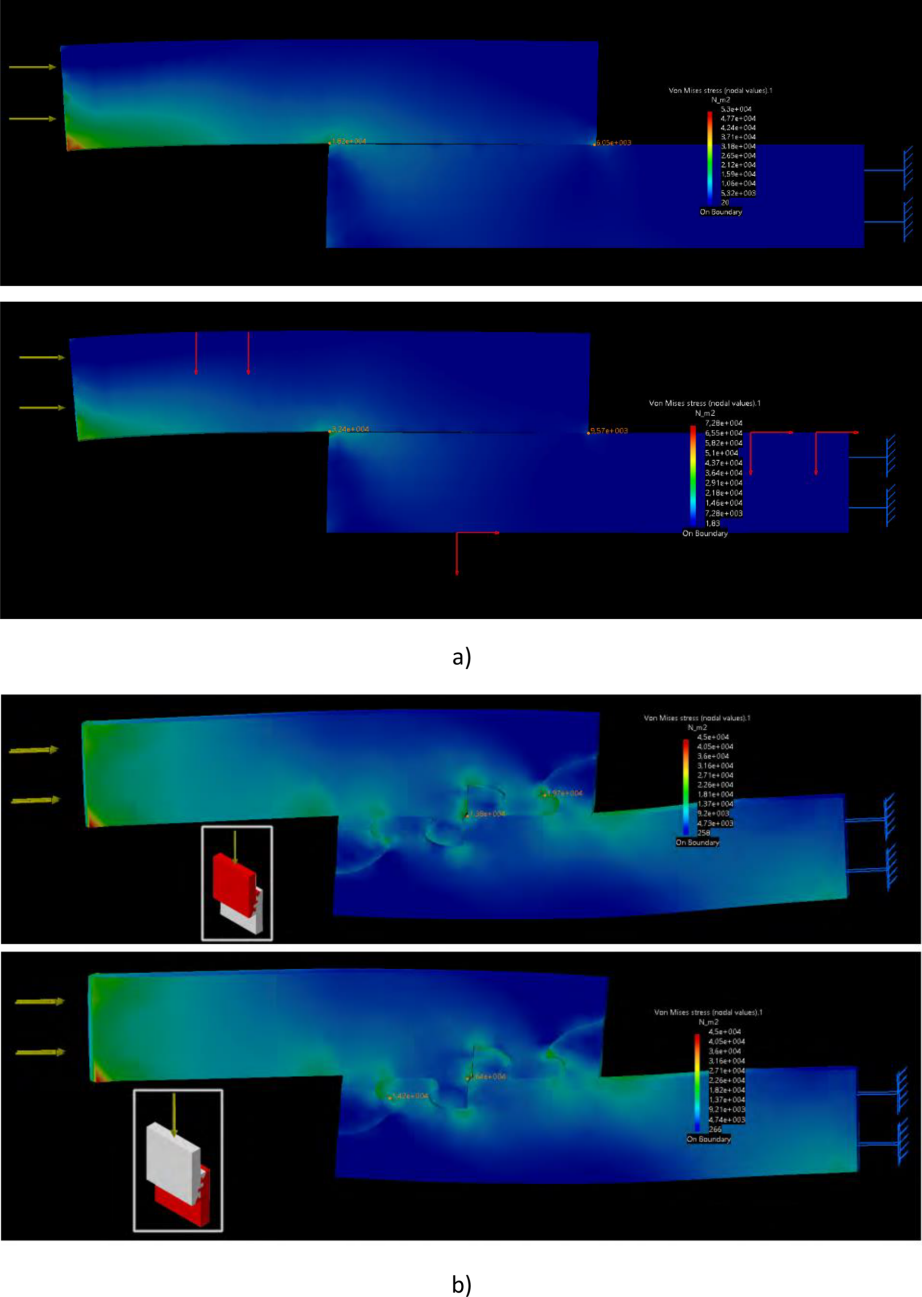
(**a**) Base: finite element analysis for 1 N compressive force: example of stress peak intensity when load is applied in compression on “base” model (on Smartfil, top; on Sakata, bottom). (**b**) V4: finite element analysis for 1 N compressive force: example of stress peak intensity when load is applied in compression on V4 (on Smartfil, top; on Sakata, bottom).

Besides, some interesting information about the stress distribution was obtained with the support of FEM resources, which provided additional insight about the failure mechanisms, about the useful application of interlocking geometries and about the interest of combining interlocks and adhesives. Figure 5 shows the simulation results, comparing base and V4 geometry, adhesively joined in all cases and simulated using the Smartfil and Sakata materials. Table 2 gathers the stress peaks registered in the simulations from Fig. 5.

**Table 2:** Summary of FEM results from Fig. 5, with stress peaks for different geometries and materials.

Geometry	Material	Stress peak 1 (Von Mises, Pa)	Stress peak 2 (Von Mises, Pa)
Base	Smartfil	1.8 × 10^4^	6 × 10^3^
Base	Sakata	3.2 × 10^4^	9.6 × 10^3^
V4	Smartfil	1.9 × 10^4^	1.4 × 10^4^
V4	Sakata	1.6 × 10^4^	1.4 × 10^4^

Some relevant aspects should be highlighted. First of all, in the case of interlocked geometries (V4), *the stress peaks appear within the interlocks, in the central regions of the joints,* where the stressed material is surrounded by supporting regions that prevent crack propagation and eventual failure. In the case of the base geometry, as in typical lap joints*, the stress peaks appear in the outer regions of the joints*, which may lead more easily to peel-off. Besides, *stress distribution along the joint is more stable in V4 than in base geometry*, for both materials. Furthermore, the base geometry is more affected by changes of material: a change from Smartfil to Sakata leads to an increase of stress peak of around 75%.

However, *in the case of the interlocked geometry, the stress peaks remain almost unaffected, compensated by a more uniform load distribution along the whole joint.* In the base geometry, the more flexible material increases bending and multiplies the stress peaks, while *in the interlocked region, the more flexible material helps to better distribute stresses within the interlocks and the joint remains almost unaffected, even with a slight *(*12–15%*)* reduction of peak stress*.

### Limitations of the study, challenges and future trends

Among limitations of the study, it is important to mention that the actual properties of the interfaces have not been modelled or incorporated to the FEM simulations, which have considered the joining or bonding regions as rigid contacts between the nodes and elements of the different parts. In the case of the first experiments, in which test probes are tested after multi-material printing, we consider that the fused deposition leads to a fusion between the two materials. In the case of the second experiments, in which test probes were printed and then joined by an adhesive, we also considered a perfect integration of the two materials across the joint. The effect of printed layers is not considered either and the materials are modelled as isotropic. In spite of being a simplified simulation approach, which may be refined in future studies, the simulations performed are quite straightforward and provide a good compromise between computational time and precision, as shown by the experimental results with the actual single lap offset tests under compression loading. Through them, the optimal geometry within a set of designs can be selected, as a result of the evaluation of stress distribution, and lead to a remarkable improved mechanical performance of the joint, as experimentally validated. In both cases the simulations help to analyse the joint and to detect regions with stress concentration, and to optimize the pile-based design of joints.

Regarding the novelty of the study, despite the fact that interlocked structures manufactured by multi-material 3D printing are already common, taking into account the references found, the study reports some innovative good practices for systematically comparing and optimizing 3D printed interlocked joints. For example, although the use of finite element modeling is already standard practice in engineering, its application to redistributing stresses within functionally graded 3D printed structures is still uncommon.

Besides, its combination with mechanical testing, employing single lap offset tests in compression load and using a bonded plane-to-plane configuration, as reference for quantifying the benefits of interlocked structures, proves useful for joint selection purposes. In our opinion, this may lead in the near future to a standardized process for evaluating 3D printed unions, in which functional gradients of properties are present. Contributing to the standardization landscape of additive manufacturing, in general, and of fused deposition modeling, in particular, is necessary for overcoming some challenges in these fields, including the systematic comparison of manufacturing results and the need for improved repeatability. Finally, the study puts forward, in a quantitative way, the benefits of combining adhesive joints with interlocked structures only achievable by 3D printing, which to our best knowledge is innovative.

Still, an important challenge for industrial deployment of these joining methods relies on the fact that the most interesting functionally graded structures and interconnections between elements of an engineering system arise when dealing with different materials families. The presented approach is applicable to multi-polymeric components obtained by multi-nozzle FDM, however technologies currently capable of additively constructing multi-material joints using more versatile combinations of polymers and ceramics, metals and ceramics, polymers and metals, among others, are uncommon. At present, it is not possible to additively process polymers together with ceramics or metals with the same technology for creating multi-material components with combinations of materials from truly different families. A remarkable exception is the possibility of lithographically combining ceramics and metals before a final sintering step leading to impressive combinations^27^.

Summarizing, results show that printing joined elements with interlocked regions can be a strategy for increasing the strength, not only of the joined regions, but also of the whole joined components, once the interlocked structures are extended to the adherend geometries. In this way, once failure initiates due to detachment of filament between printed layers, the presence of interlocks may limit crack propagation. In addition, the beneficial synergic effect between interlocking geometries and adhesives is demonstrated.

## Conclusions

In this research and experimental study, the team of authors has explored the combined application of modelling and ALM for creating and testing materials with interlocking structures, aimed at reducing the stress concentration along the edges of a typical lap joint. The effectiveness of this approach has been researched and discussed by means of modelling, ALM, through multi-material fused deposition modelling and testing of thermoplastic test probes with a diversity of interlocking elements.

*The combination of suitable mechanical interlocking and adhesive bonding has once more demonstrated to be the best option to maximize the mechanical strength of joints.*

Future studies will be linked to translating these joining strategies to other types of materials, including photopolymers, ceramics and alloys, thanks to the use of alternative ALM techniques, some of which are currently under development.

## Data Availability

The raw/processed data required to reproduce these findings cannot be shared at this time due to technical or time limitations. They will be made available before eventual acceptance and publication through an open repository by the participant institutions.
